# Mixed reality for midwifery students: a qualitative study of the technology’s perceived appropriateness in the classroom

**DOI:** 10.1186/s12909-025-06919-z

**Published:** 2025-03-05

**Authors:** Linda Wike Ljungblad, Dooley Murphy, Hannah Elisabeth Fonkalsrud

**Affiliations:** 1 University of South-Eastern Norway (USN), Centre for Women’s, Family and Child Health, Box 235, Kongsberg, N-3603 Norway; 2 Laerdal Medical, Njalsgade 19D, Copenhagen, 2300 Denmark; 3 University of South-Eastern Norway (USN), Box 235, Kongsberg, N-3603 Norway

**Keywords:** Education, Foetal stages, Midwifery, Mixed reality (MR), Pedagogy, Peer-to-peer learning, Teaching, Technology, User experience (UX), Virtual reality (VR)

## Abstract

**Background:**

Virtual reality and mixed reality have shown great promise in training and education across a range of professional and pedagogical domains. The perception of such technologies by midwifery students remains an underexplored area of study.

**Methods:**

Thirty-three MSc midwifery students received a demonstration of a proof-of-concept mixed reality lesson about the foetal descent during labour. Twelve students were subsequently interviewed about their experiences, and thematic analysis was used to analyse the qualitative dataset produced by the interview transcripts.

**Results:**

Analysis found [1] that mixed reality was viewed by the students as a valuable novelty which facilitates new insights while scaffolding prior learnings [2], that mixed reality was postulated to gel well with other learning methods and modalities such as simulation-based training, and [3] that while mixed reality was intuitive or easy to use, adaptable or customisable content should be a key consideration in immersive lesson design.

**Conclusions:**

The study concludes that mixed reality can be a valuable supplement to existing teaching methods and tools. Students expressed optimism about mixed reality’s potential to enhance educational outcomes. While it cannot replace dialogue with a qualified instructor, mixed reality may be well suited to facilitating peer-to-peer learning.

**Clinical trial number:**

Not applicable.

## Background

Virtual reality (VR) and mixed reality (MR) have shown great promise in training and education across a range of professional and pedagogical domains. Examples range from forensics to engineering [1–10].

VR and MR are delivered through headsets like the Microsoft HoloLens 2 or the Meta Quest 3. These technologies provide access to immersive, spatialised, interactive 3D content. Their “room-scale” and embodied nature allows students to engage with complex material in ways that traditional teaching methods and media cannot offer [11]. For instance, rather than studying static anatomical diagrams, trainees in urogynaecological surgery could use VR to navigate larger-than-life-sized models of the pelvic floor as if exploring a cave [12]. Dental anaesthetists, using software that simulates treating a patient, could train individual tasks while simultaneously practising high-level procedures and decision-making [13]. Or in a virtual birthing suite, midwifery-related competencies such as educating expectant mothers or using closed-loop communication with colleagues could be trained and evaluated in a low-dose, high-frequency manner.

Low-dose, high-frequency training (LDHF) is an effective way of acquiring and maintaining maternity-adjacent and other healthcare competencies [14–18]. Administering mixed reality midwifery education in a low-dose high-frequency fashion could increase student engagement and knowledge retention while lowering logistical barriers to simulation-based training. The following section elaborates mixed reality’s potential benefits by describing possible use-cases, then explores the desirable psychological effects of MR in educational contexts.

### Enhancing midwifery education with mixed reality

Mixed Reality (MR) could be used to enhance at least four aspects of a given perinatal care curriculum. First, there is knowledge acquisition, sometimes called the cognitive component. Annotated 3D visualisations of foetal and maternal anatomy could serve as an immersive augmentation of traditional teaching materials, comparable to an interactive textbook. Second, there is task training. Psychomotor skills that are usually practiced on physical task trainers could be guided by virtual overlays and voiceover narration. For instance, an MR birthing simulator could show the position and presentation of the foetus in an obstetric manikin’s birth canal, with students able to see and hear about the outcome of their interventions in real time. Third, there is procedure training. If we think of procedure training as comparable to task training but with additional steps, context, and stressors, MR could add to the realism and transferability of a session by bringing a manikin to life. For example, by adding an AI overlay that gives the physical birthing simulator emotions, expressions, and the ability to voice concern if inappropriate actions are taken. And fourth, by the same token, full-scale medical simulations could be made yet more immersive, with the introduction of MR (or VR) reducing the amount of consumables required (e.g. artificial blood or silicone perinea).

Beyond what is possible in terms of modifying classroom or sim room experiences, let’s note the broadly beneficial psychological effects afforded by VR and MR that occur independently of learning content. Two such effects, which are conceptually separable but functionally interrelated, are *presence* and *engagement*.

### Relevant concepts and similar studies

Presence is the perceptual illusion of being in a real environment or of virtual objects being real. Presence in a spatial scene is often defined as “the feeling of being there” [19, 20]. In the case of MR, presence refers primarily to virtual objects appearing as part of the user’s physical space. Psychologically speaking, presence is automatic and unconscious insofar as one cannot wish or will the illusion away. This is true irrespective of whether one is paying attention to or “believing” what one’s eyes are reporting. For example, if a student dons an MR headset and sees a lifelike virtual mother reclining in a birthing bed, we would say that the technology has induced a sense of social presence, an illusion of co-location. Studies suggest that the student is practically guaranteed to exhibit realistic “low-level” social behaviours (e.g. establishing eye contact with the mother) regardless of whether they truly believe her to be there [21–24].

An optimal level of presence—whether social or spatial—enhances learning. Makowski, Sperduti [25] ran a study in which factual memory, emotion, and presence were measured in film viewers. They found that the latter construct mediated the former two, concluding that a sense of presence (in their case, a feeling of “being there” in a movie’s fictional environment) supported memory encoding.

Krokos, Plaisant [26] measured participants’ ability to recall the contents of mnemonic “memory palaces” in a high-presence VR condition versus a low-presence desktop computer condition. Participants were instructed to navigate a virtual environment and to remember (also subsequently recall) the locations of faces and numbers displayed in picture frames. They found that those who internalised and recalled information in the immersive VR condition did so more confidently and accurately than those in the desktop computer condition while also skipping fewer items. This informed the authors’ conclusion that presence enhances both information retention and recall.

Oppositely, in similar experimental setups employing memorisation tasks, both Ochs and Sonderegger [27] and Makransky, Terkildsen [28] found that being in VR seemingly decreased participants’ ability to achieve learning outcomes. They hypothesise that this is because the VR participants were cognitively overloaded. Hence while presence in a virtual or mixed reality environment is generally desirable, overstimulation can have a negative impact on memory. It is important, then, not to overwhelm novice VR or MR users. For newcomers, simply wearing a headset can be mentally taxing regardless of task or activity. This may make MR more promising than VR for medical education: Instead of placing the learner in an immersive but unfamiliar environment, a select few virtual objects can be brought into the mostly real (but mixed) reality classroom, minimising the risk of sensory or cognitive overload.

Separately from presence, engagement describes a sense of intense interest in a virtual environment [29, 30], its learning content [31], or both [32]. A function of “trait absorption”—that is, an individual’s tendency to become fascinated by something [33]—engagement is self-evidently beneficial to learning insofar as it presupposes that the learner is focused on the subject matter, with distractors being tuned out of conscious awareness. Though the psychological construct of engagement is defined and operationalised slightly differently in educational psychology compared with media research, its implications are clear: An engaging lesson is always preferable over an unengaging one. Given its relative novelty, MR could stand to make certain aspects of midwifery education (such as maternal and foetal anatomy or physiology) more engaging to students who may otherwise prefer the interpersonal aspects of perinatal care over biological topics.

Before segueing into the present study’s Methods section, let’s briefly outline the types of relevant research that are common at the time of publication. The most widely represented genre of MR study in healthcare education is the narrative or scoping review [7, 34, 35]. These studies generally describe a given technology or intervention’s possible affordances and drawbacks without getting “hands-on” with a specific device or software application. Narrative or scoping reviews are hence somewhat speculative and comment on the nature of technology often without discussing concrete features. Accordingly, narrative or scoping reviews do not, in the strictest sense, provide new or primary data with which other researchers might corroborate or contrast their own findings.

Similarly, systematic reviews and/or meta-analyses [10, 11, 36–38] qualitatively or statistically synthesise others’ findings into new second-order data. While systematic reviews and/or meta-analyses do not produce new data by introducing students or educators to the tools with which they may soon be working, this type of study is at least less conjectural than narrative or scoping reviews. Meta-analyses, of course, aggregate findings to validate effect sizes, providing an indication of whether a given result is meaningfully replicable and ultimately indicating what can be considered scientific canon.

With the creation of valid and reliable new data in mind, the most desirable type of MR study at present is one employing a classical experimental setup. That is, a randomised, between-groups design in which some students are exposed to an MR intervention, others are not (and are instead assigned to a control group that is given textbook material or educational videos instead of MR), and the groups of students’ performance compared. This type of study is typically best equipped to answer the question, “does intervention X produce result Y?”, with the null hypothesis that there are no advantageous effects ideally being disproven [39].

While randomised controlled trials are the gold standard, in the context of healthcare education they are often best preluded by a pilot study testing a technology’s goodness-of-fit or appropriateness for the classroom, birthing room, or other locale. Therefore, following an example set by others [39, 40], we believe that before quantifying the efficacy of a given educational–technological intervention, it is thoughtful to qualitatively explore whether students find the new paradigm approachable, agreeable, intuitively usable, and so on. Such is the purpose of this study.

## Methods

### Aim

To explore whether and how midwifery students benefit from learning about foetal stages in labour by using immersive, interactive 3D visualisations displayed in the Magic Leap 2 mixed reality headset.

### Setting

Participants (*n* = 33) were recruited from a cohort of first-year midwifery students at a Norwegian University during a period of scheduled simulation training held prior to clinical placement. In Norway, a BSc qualification in nursing is a prerequisite to enrolment for a Master’s degree in midwifery, in accordance with the Bologna Process for standardised degrees [41, 42]. The program spans two years and strikes a 50–50 balance between campus-based education and clinical practice, emphasising high-fidelity, team-based simulation for teaching obstetric procedures and managing emergencies as well as normal births.

### Study design

The study took an explorative, qualitative approach comprising four distinct stages.

First, participants completed a short pre-exposure questionnaire through which they reported basic demographic information and their level of user experience (UX) with various technologies. The purpose of this stage was simply to determine if there were any prospective participants with an anomalous lack of experience with technology, or if there were outliers in terms of age.

Second, participants were exposed, two at a time, to the collaborative MR learning activity, the content of which is soon described in the Exposure section.

Third, participants completed a post-exposure questionnaire that evaluated the MR lesson’s usability—and the wider technological experience—per criteria such as comfort, (in)convenience, performance, and intuitiveness. These Likert data are not discussed in the present paper. Crucially, the post-exposure questionnaire had students rate their overall experience as positive, neutral, or negative, which allowed us to select interview participants based on their attitudes towards MR (for purposes of representativeness). In other words, though the majority of students rated the experience positively, we took measures to include the voices of the two students who felt ambivalent about it, as well as the one student who flatly disliked MR. Thus, from the pool of students who responded positively, nine were randomly selected to join the above-mentioned three neutral and negative respondents in sharing further reflections.

The fourth and final stage of the study entailed three midwifery lecturers individually interviewing a total of twelve students about their positive, neutral, and negative perceptions of MR. Interview data were then analysed using thematic analysis [43, 44]. Themes were reached by way of codes that were grouped into categories before being reduced or condensed and relabelled.

### Participant

Thirty-three midwifery students were present in the classroom and thereby included in a convenience sample universe. All who completed the pre-exposure questionnaire identified as Norwegian and female. Four respondents were 22–25 years old. The age brackets 26–29, 30–33, and 34–37 each had eight representatives. Five participants were aged 38 or above. In summary, about three quarters of participants (74%) were aged between 26 and 37, which is perhaps typical of midwifery programmes in Norway but slightly higher than the average age of trainee midwives internationally.

Twenty participants had never previously tried any headset-based technology (VR or MR). Twelve had tried VR once or twice. One respondent reported having spent a number of hours playing VR games at home. None had tried MR prior to the exposure.

Regarding other digital screen technologies (e.g., smartphones, tablets, and computers), about half the respondents considered themselves “experienced but not very confident”. About a quarter self-described as “experienced and confident”. Age showed no correlation with self-reported confidence or ability with technology.

### Exposure

Students entered the classroom in groups of six and, two at a time, put on the supplied Magic Leap 2 headsets. The Magic Leap 2 is an “optical see-through”-type mixed reality device, meaning users perceive their physical environment through transparent glass lenses *into* which virtual content is projected, overlaying and augmenting reality. This is noteworthy insofar as the Magic Leap 2’s see-through displays eliminate the risk of cybersickness—a type of motion sickness that is sometimes associated with screen- and video-feed-based VR and MR headsets.

**Fig. 1:**
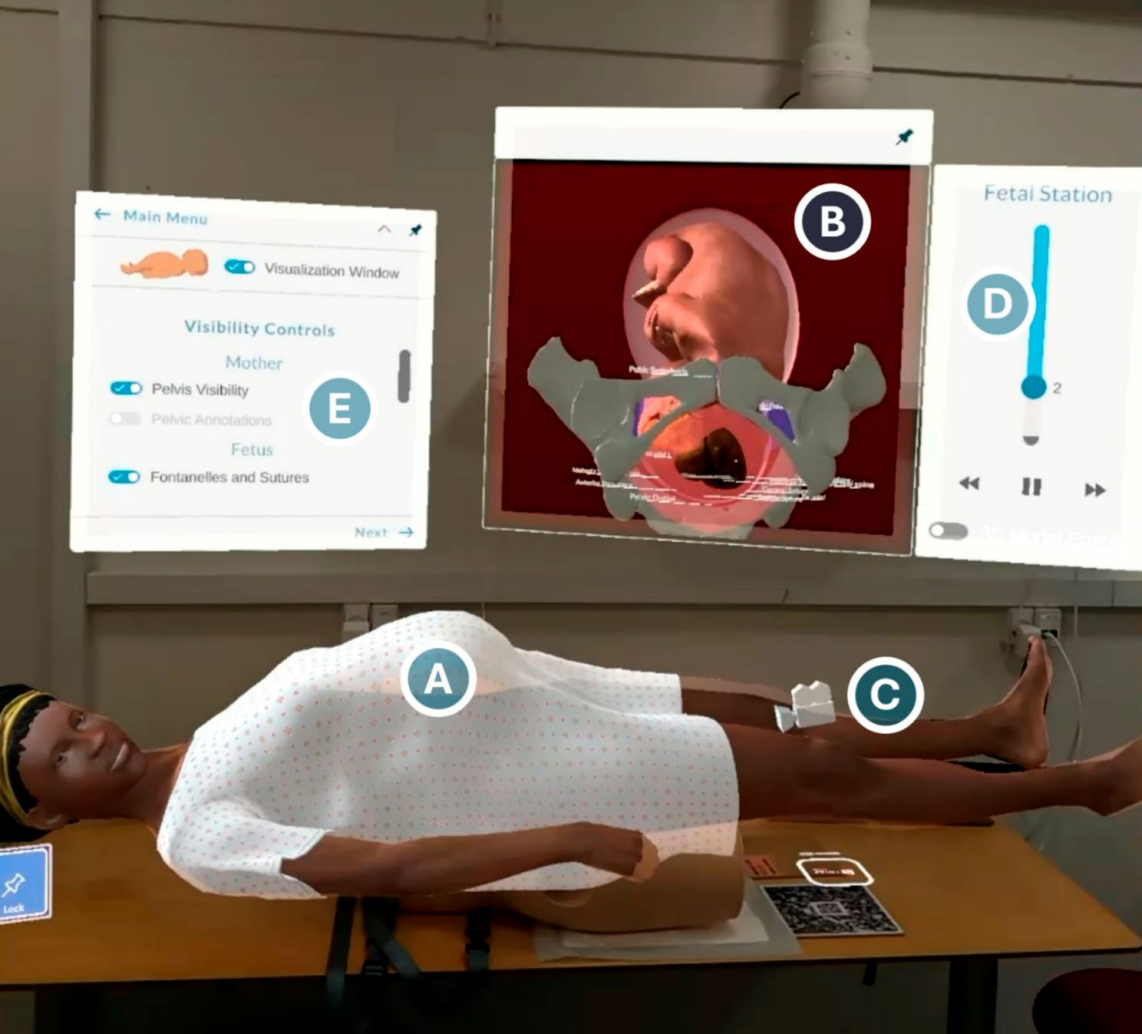
An illustration of what the students saw. A blend of virtual and real-world elements—albeit in a different physical environment. (Pictured: A test room in Copenhagen.) For explanations of the letters’ significance, see body text

A designer from Laerdal Medical (2nd author DM) had set up the session in advance. Upon donning the headsets, participants saw (A) a clothed mother laying supine; (B) a large interface window containing an interactive 3D visualisation of an *in utero* foetus and maternal pelvis; (C) a manipulable camera for changing the viewing angle of the *in utero* “x-ray” visualisation; (D) a slider for advancing the displayed foetal station and voiceover narration; (E) additional controls for showing and hiding annotations, labels, and highlights (e.g. coloured planes to indicate the pelvic inlet and outlet, a line drawn between the ischial spines, labels denoting sutures and fontanelles on the foetal cranium, etc.); and also, off-screen but available, a drop-down menu for changing the foetal position between three presets. See Fig. 1.

Students were instructed to first familiarise themselves with the MR app’s controller and spatial user interface, then to proceed through the foetal descent lesson. A typical exposure went as follows. With their respective controllers held in the dominant hand, each pair of students first practised grabbing and moving the 3D camera to alter the virtual viewing angle of the *in utero* visualisation. They were advised to corroborate and discuss what they were seeing, doing, and hearing to ensure that the shared MR visualisations were synchronising correctly across the headsets and to underscore the collaborative, social nature of the learning experience.

Once comfortable with the app’s controls (which generally took about 3 min per pair), students began to follow the progress of the foetal descent. One student would press a “Play” button which activated a segment of voiceover narration and an accompanying animation of the foetus’ descent down the birth canal. The metric used was − 5 to + 5 station, corresponding to eleven pieces of voiceover narration in total. Students generally followed their instructors’ advice to pause between each foetal station and explore the interactive 3D visualisation to ensure that they’d grasped the location and relevance of anatomical landmarks and physiological processes mentioned in the audio description. The audio description and text labels were in English, with the possibility of a live Norwegian translation from a midwifery instructor upon request.

Most pairs of students concluded the exposure after listening to all eleven pieces of narration, though some continued to explore the app until they felt sure they’d tried each and every feature. (For instance, students weren’t told to change the foetus’ position from, e.g., occiput anterior to occiput posterior or toggle annotations on and off—they were left to discover this themselves.) An average exposure lasted 15–20 min per pair of students.

After all students had experienced the MR foetal descent, post-exposure questionnaires were administered. At this point, two students either left the classroom or failed to submit their questionnaires. Hence the total number of post-exposure questionnaire responses (and by extension, the pool from which twelve interviewees were selected) was ultimately 31.

### Qualitative interviews

Twelve individual, semi-structured interviews were conducted following the MR exposure. Students were granted anonymity in submitting their post-exposure questionnaire responses and allowed to opt-out of the interview selection process if they so desired.

Three midwifery lecturers employed at a Norwegian University completed four interviews each with the quasi-randomly selected participants. As mentioned previously, the two students who reported their perception of the MR experience to be neutral and the one student who rated it negatively were automatically included in the interview pool. The remaining nine were randomly selected by an arbitrary identification number (and not by name) by the representative from Laerdal Medical, to eliminate any possibility of instructor–student bias.

**Table 1:** Semi-structured interview guide

1. How did you experience using mixed reality (MR) technology to learn about the foetal descent during childbirth? 2. Was it useful for you to see foetal rotations visualised with the help of a hologram? If so, in what ways? 3. Did the MR visualisations conflict in any way with what you expected to see, or what you have previously learned? Please go into detail. 4. Did the voiceover narration conflict in any way with what you saw, or what you expected to hear explained? If so, how? 5. How do you feel about the level and quality of the educational content? 6. How did you experience MR compared to other ways of learning, such as 3D animations on YouTube or cross-sectional diagrams in textbooks? 7. How did you experience the working method of discussing in pairs (peer-to-peer) while exploring the MR holograms? 8. Is there anything about this learning experience/paradigm that you would change? Feel free to mention both MR content and the way it is presented in the classroom. 9. Did you experience any challenges that affected your learning? If so, what? 10. Do you have other comments or suggestions?	

The interviews were conducted on-site at the university, and students were encouraged to speak freely; to candidly share and elaborate on their experiences of MR. They were not asked about their midwifery or medical knowledge—only about their experience of using the novel headset-based technology in learning foetal rotations. All interviews were conducted in Norwegian (the students’ native language) to create a comfortable environment that would foster meaningful reflection, openness, and sincerity. A semi-structured interview guide was used (see Table 1), and follow-up questions were asked when there was a perceived need for the students to clarify or elaborate upon their comments or ideas.

### Ethical considerations

This exploratory pilot study was planned according to the Declaration of Helsinki: Ethical Principles for Medical Research in Human Subjects [45]. Approval to collect and store data is given from Sikt (the Norwegian Agency for Shared Services in Education and Research), reference nr. 999,942. The data collected are stored on a safe server at the university, and the alphanumeric codes used to identify the participants are kept separately to ensure anonymity. Written information and consent to participate in this study were given and collected prior to participation. No remuneration or other benefits were given to the students for participation, and likewise there were no negative consequences for declining to participate in any part of the study. No benefits were exchanged between USN and Laerdal or their representatives. Prospective participants were informed that the study would be written-up and published as a scientific paper, and that quotes may be used anonymously. Students were not exposed to any material not appearing on the curriculum for which they were enrolled.

### Data creation and analysis

Qualitative data were created and analysed using thematic analysis as described by Virginia Braun and Victoria Clarke [43, 44]. Data creation and analysis consisted in six phases.

**In phase one**, we (LWJ and HF) became familiar with the material from which the data would be drawn. First, for the sake of accuracy and convenience, we read and corrected our automatically generated transcripts of the interview audio recordings before sharing and getting to know each other’s. This allowed us to get acquainted with the depth and breadth of the content from which our data (or units of analysis) would be fashioned. When we re-read all transcripts, we read the source material actively, as the methodology suggests, and searched for meanings or patterns in the transcripts that were (literally) highlighted. Notes and other annotations were also added to the paper transcripts at this stage.

**In phase two**, we created an initial list of ideas about what the source material entailed *in toto* before identifying initial codes to serve as fundamental data points. We made a table documenting where the data extract was to be found in the transcripts on the left-hand side, and the initial code—a quoted word, short expression, or paraphrased snippet—was written on the right-hand side of the table.

**In phase three**, we printed the code table from the latter phase and cut out each row (again—literally, using scissors). These were sorted into preliminary categories. We looked for patterns, qualitative similarities, and differences in the data. We were open-minded, and some prior initial codes were changed during this phase. We ended up with twelve categories to be further reduced and refined into three themes.

**In phase four**, we combed the categories for nascent themes. We searched for internal homogeneity in each category by re-reading its constituent codes and finding coherent patterns grouped around either topics or sentiments. Nine categories were thus reduced, merged, or discharged.

**In phase five**, we defined and refined the themes, searching for the essence of each. We wrote a story about each theme by way of its contributing categories. This transpired to be an iterative process: Themes changed names, categories were further collapsed into one another, and extracts (codes) even moved positions.

**In phase six**, we wrote a final report of the analysis for each theme in a manner that described them concisely and non-repetitively. (I.e., non-redundantly.) Ordinarily, the term “mutually exclusive” might pertain here. However, our three final themes were not designed to be exclusory of one another—each was intended to describe a different facet or dimension of the students’ collective and individual experience. Each theme’s persistence through this final phase was justified by its relation to the study’s overall aim: To qualitatively explore whether and how MR technologies benefit midwifery students learning about foetal descent during labour.

## Results

Three themes housing nine subthemes were produced by analysis, as illustrated in Table 2 below and elaborated in prose in subsections thereafter. An overarching theme was created to unify the other themes, which will be presented at the end of this section.

**Table 2:**
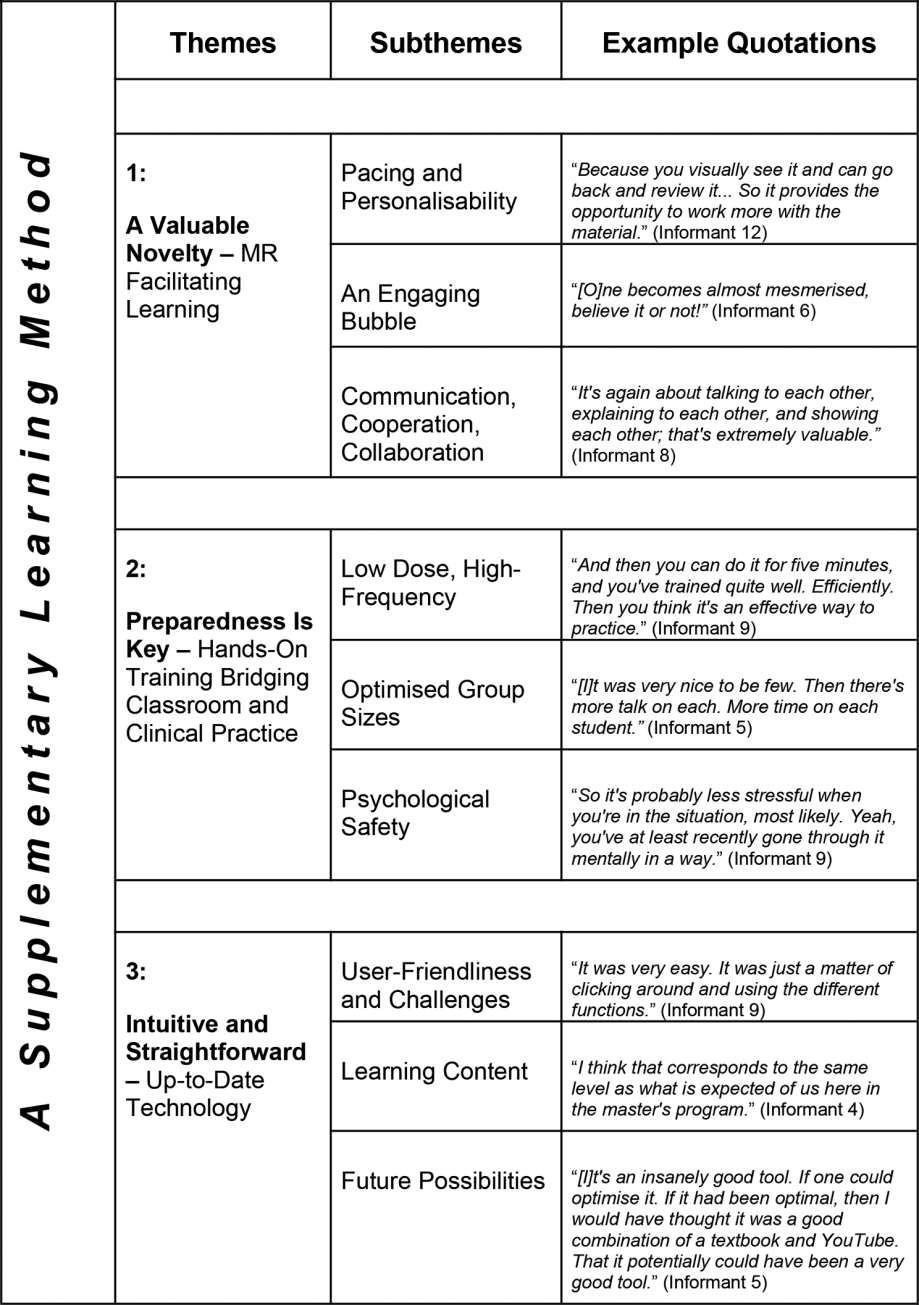
An overarching theme and three themes covering nine subthemes in total

### Theme 1: A valuable novelty – MR facilitates learning

A majority of participants described an overall impression that MR contributed to supplementary and repeated learning; that it could illuminate, cement, or galvanise ideas acquired in other stages of the students’ learning journey (for example, during classroom lectures, through textbook-based study, or while watching computer-generated videos of the foetal descent.) Several students alluded to the worthiness of the stereoscopic (“3D”) foetal–uterine visualisation, commenting that its realism and scale surely deepened their visuospatial understanding of maternal–foetal physiology and the continuum of labour’s progress. Some students mentioned that “a light was lit,” with MR serving as a “new tool” to reinforce knowledge acquired previously in other domains; they experienced a beneficial confirmation of their preexisting theoretical understanding.

For individuals like myself, who lean more towards practical learning than theoretical, it was highly beneficial to visually observe the rotations on the inside of the pelvis in a way, like rather than solely reading about it. Visualising the rotations provides a more tangible understanding, offering additional mental hooks to anchor the concept. (Informant 4)

Others experienced MR technology as an engaging and even entertaining learning method that somehow felt more “alive” than computer-generated visual content displayed on “flat” (i.e., non-stereoscopic) screens such as laptops or tablets.

It adds a bit spice, making it somewhat enjoyable. (Informant 8)

One student held that MR technology did not contribute to any new learning possibilities for her and that she could find the same content in videos on YouTube. Another highlighted a lack of tactile sensations.

### Pacing and personalisability

A big part of the description of MR as facilitating learning included the students’ experience of the lesson as adaptable or customisable. They mentioned that controlling or choosing the rate of information-dispersal by individual needs was central to the lesson’s perceived success or efficacy. In plain terms, students found it helpful that the MR lesson was advanced one foetal station at a time and that the voiceover narration paused between each step, giving them an opportunity to discuss, reflect, understand, and explore. Several reported that this enabled them to go back and forth at their own pace and repeat segments as necessary.

And it was really nice that you could go in and adjust and control the speed of the movements in the pelvis. That way, you could see each of the movements in detail. And being able to fast forward and rewind, as well as rotate the pelvis to view it from different angles, was very beneficial. (Informant 4)

That the visualisation’s camera angle could be modified; steps repeated; annotations or visual layers toggled on and off, etc., all contributed to a sense that the MR solution’s customisability is a pillar of its valuable novelty status.

Being able to move forwards and backwards, rotate, see where the baby was, which area of the pelvis, and so on, is indeed a good alternative for learning. (Informant 1)

Overall, the MR lesson seems to have given students a sense of autodidactic agency—a consequence of MR’s interactivity—that makes the paradigm feel more “versatile” than other audiovisual learning materials such as videos or podcasts.

### An engaging bubble

A second subtheme nested under “A Valuable Novelty – MR facilitating learning” relates to a feeling of engagement in the MR environment. Students talked about being “inside the bubble,” in their own world, or of participating in a video game; of being transported cognitively.

Perhaps one immerses differently when wearing the glasses… You can’t fully… [B]ut you become more absorbed when it’s just yourself and the scenario… You’re almost in a video game, in a way. (Informant 9)

While some students engaged with the MR lesson because sights and sounds appeared before them, others commented that they enjoyed it because no roleplay was expected from them. They were able to detach and consume the content individually without actively roleplaying as one would have to in typical medical simulation training.

You don’t need to play a role to the same extent as you do in a simulation scenario. That’s something many struggle with and find quite uncomfortable. (Informant 9)

Reportedly, this made learning less performance-focused, which helped students better recall and remember: They did not feel the pressure of playing a role, and hence had an easier time internalising and/or contextualising the information presented. It is worth noting that one student said that she preferred books and handwriting. Just as the majority enjoy the novelty of the technological solution, it is reasonable that some will find similar affective appeal in the tactile romance of analogue note-taking and page-turning.

### Communication, cooperation, collaboration

The last part of our first theme, “A Valuable Novelty – MR Facilitating Learning”, regards the students’ experience of collaboration. Working in pairs during the MR session gave them the opportunity to widen their pedagogical potentials by adding focus to communicative and cooperative teaching and learning. Acting as co-instructors to one another in a peer-to-peer learning method, pairs of students were challenged to become both drivers and passengers.

Perhaps you hadn’t even considered it alone, but in collaboration with someone else, you gain more… opportunities and greater learning potential. Then you have a broader, what should I say, focus? (Informant 3)

They experienced this by co-working, co-questioning, co-explaining, co-illustrating, co-agreeing, and co-understanding. While this peer-to-peer learning aspect of the MR solution was practically a necessity (owing to the suboptimal campus Wi-Fi speed producing desynchronised MR visualisations), it transpired to be advantageous: Students roundly appreciated having to confirm that they were seeing and hearing the same things at the same time; that they’d noticed the same anatomical landmarks (e.g. located the ischial spines); that one student’s manipulation of the virtual camera angle was visible and acceptable to the other, and so on. This need to constantly check in with one another reportedly triggered and scaffolded among students new processes in thinking, resulting in richer reflections compared to studying alone.

### Theme 2: Preparedness is key – hands-on training bridging classroom and clinical practice

In striving to attain readiness for clinical practice, students emphasised the significance of experiential learning through hands-on activities. They underscored the value of the MR solution’s “almost reality” (“almost lifelike”) quality, which they described as offering an immersive yet safe approximation of reality.

It’s nice to try in almost reality without it being reality. (Informant 5)

They noted that the virtual mother at the heart of the MR simulation could even be construed as more lifelike than traditional plastic medical manikins. Participants highlighted the opportunity to gain tacit knowledge through simulated scenarios prior to encountering real-life situations. They found MR simulations to be a practical approach that enabled them to experiment and learn from mistakes in a secure setting; a “safe space”, or even safer space than the simulation room—perhaps because it was easy to imagine the lesson without the invigilating presence of an instructor or facilitator.

One can practice certain things, which makes you feel a bit more confident when you go out into practice and do things for the first time. It’s also a reasonable way to practice and learn from mistakes. (Informant 7)

### Low-dose, high frequency

The participants discussed the importance of increasing the frequency and digestibility of hands-on training opportunities for better preparation in clinical practice. In plain terms, they found “bite-sized” lessons preferable.

So instead of being in a simulation room, you have the visuals in the room or wherever you’re sitting. So it’s very positive in that regard. (Informant 10)

**Fig. 2:**
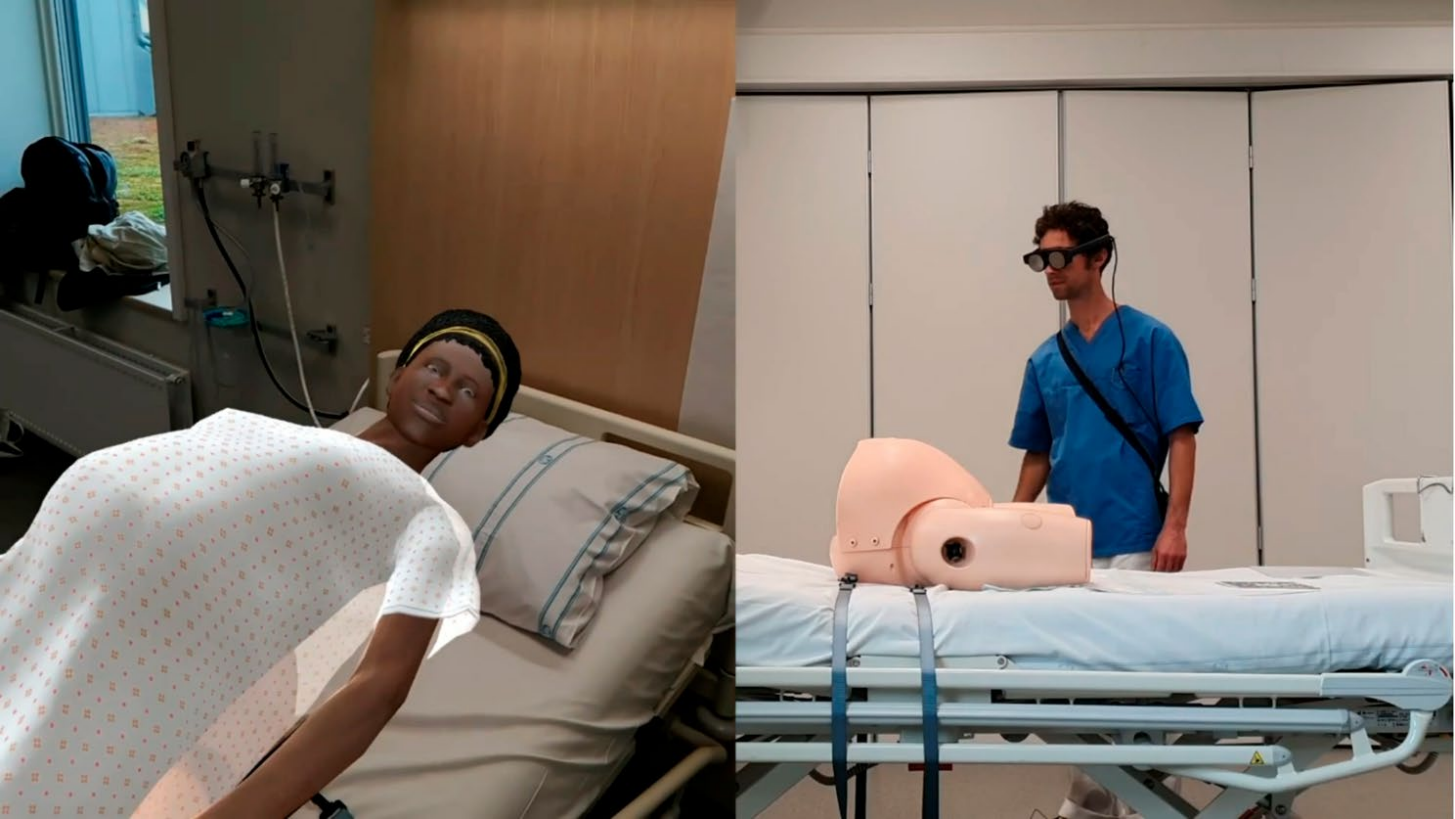
Illustrations of technology used in this study. Although the students were not shown the software in the context of simulation-based training, several found it easy to imagine how conversing with an AI mother and using MR in combination with simulators or manikins could be beneficial for increasing confidence and competence. Pictured on the left is an actor’s view of the MR mother overlaid on top of a low-fidelity simulator. On the right: A view of the simulation room

They described MR as uncomplicated and emphasised its ability to provide effective, short-form, repeatable, and low-cost training sessions. Note that although the lesson demoed was a “textbook-style” lesson focusing on knowledge acquisition (a “cognitive” component of midwifery, to use our prior terminology), the students seemingly found it easy to imagine how MR experiences could be adapted to simulation-based procedure training or even fine psychomotor skills training, emulating real-life birthing scenarios and emergencies. (See Fig. 2.)

MR as a learning method was suggested to be useful not only for students but also potentially for experienced midwives.

### Optimised group sizes

For conducive learning sessions, students proposed small groups of four to six participants (rather than working pairs, as in the exposure). Optimal group sizes were speculated to consist of more than two students but fewer than ten.

Not too many though, but it could have been four or so, but not up to ten. I think that was too much. But it was very nice that you could discuss…(Informant 6).

Smaller group sizes were thought to improve activity and decrease limitations so the students would feel more comfortable, and to offer time-efficient learning sessions.

Another suggestion to improve peer-learning was the use of a TV or large screen to display, via casting or streaming, a duplicate of an MR user’s first-person perspective. This would be visible for non-participant observers in the room. Ideally, every student in the group should be able to wear an MR headset. However, as mentioned, comfort or so-called cybersickness can be a concern with the more affordable and widespread style of MR headsets such as the Meta Quest 3. Yet either way, we can informally observe that even when the risk of motion sickness is nil, some group members will always decline to wear a headset. Perhaps this is for hygiene reasons, because they’re worried it will smudge their make-up or mess up their hair, because they’ve had bad past experiences with VR, or because they don’t want to feel self-conscious by being the centre of attention.

### Psychological safety

Students expressed that being aware of the bodily sensation of stressful situations and learning to manage them in simulated scenarios aided their preparation for clinical practice. Through mentally rehearsing various scenarios, participants believed they could reduce stress levels and enhance confidence through repetition. This is, again, despite the fact that the lesson demoed for students was not a simulated birth per se, and certainly not an obstetric emergency requiring acute management. The ability to effectively handle situations, facilitated by the use of MR simulations, was perceived as a means to achieve learning outcomes in the progression toward midwifery expertise; the mastery of competencies.

### Theme 3: Intuitive and straightforward – an up-to-date technology

Mixed reality technology was expressed to be beneficial, innovative, exciting and simultaneously intimidating or even scary.

If we become more accustomed to it, it will probably become more normal for us, than now when it’s new and exciting and scary at the same time. (Informant 1)

The students were clear about that such technology is “up-to-date,” and they were excited to be introduced to it early in their midwifery education, as they already know of MR and VR use in other medical and healthcare settings.

A very good setup for further learning. It’s in line with the times. It becomes more live in a way. (Informant 7)

The students underscored the importance of repetition to reinforce learning outcomes. Their excitement additionally included a focus on incorporation of such innovative MR technology within the relatively narrow field of midwifery. Although, as mentioned, one student mentioned the MR session not to be ground-breaking.

### User-friendliness and challenges

MR was described overall as intuitive and user-friendly. Participants found it easy to explore the immersive visualisations by using the software’s window and “laser pointer”-based user interface via a handheld controller, and they quickly became confident in navigating and interacting. The students were generally very positive about MR in terms of the possibility of seeing the real world “through” the headset’s fully transparent lenses, which gave them better spatial awareness, greater prerequisites to collaborate with peer students, and negligible sensations of dizziness compared to VR. They found the equipment comfortable to wear and that they easily accessed the “MR mood.” Participants suggested a five-minute introduction video about the practical use and the possibilities of the software, provided prior to the session, to support learning outcomes.

It would have been nice to get a little demo video for five minutes just showing a bit what things meant… five minutes, like that, and then you can do it. (Informant 6)

Several students talked about technical challenges at different levels such as poor Wi-Fi and battery capacity as well as hard- and software bugs. Such hiccups confused a few students who felt that the uncertainty introduced disrupted the flow, decreasing learning outcomes.

### Learning content

Participants reported the learning content to be consistent with midwifery theory and that it did not conflict with their expectations and level of knowledge. Some students experienced the MR session as beneficial way to recall and refresh knowledge, skills, and competencies. A few found the level of content to be too basic and they requested a possibility to choose between different degrees of difficulty.

That perhaps I had imagined there would be a bit more to strive for, in a way. That I would have raised my level of knowledge better from today… (Informant 5).

Norwegian language was discussed to be more efficient since the students found deeper interactions and expressions of feelings harder in a non-native language. They mentioned a lower degree of learning outcome as a consequence.

It’s a bit challenging as I have to switch my brain to English language… It becomes a bit more difficult to learn” (Informant 3).

Participants also talked about confusion stemming from the different descriptive terminology used to describe foetal stations internationally (–5 to + 5, with integers denoting centimetres) versus in Norwegian (wherein foetal station bears a flexible descriptive relation to the pelvic bones).

### Future possibilities

All participants had an impression of MR as a beneficial tool with pedagogical potential. They were eager to mention future possibilities such as a potential brickwork/puzzle to build a pelvis with names of the different parts (skeletal, muscular, etc.), performing a Leopold’s manoeuvre, and turning markers (e.g. pelvic inlet/outlet) on and off. In addition, many students requested to be able to see the labour process continued until delivery, and birth in varied positions. When it comes to pathology, acute procedures were described suitable to practice in MR. They mentioned in particular shoulder dystocia, newborn resuscitation, postpartum haemorrhage, and (pre)eclampsia. Participants believed such training tool would be as useful for students as for experienced midwives.

I think it would be useful, whether you’re a student or a practising midwife. (Informant 2)

### Overarching theme

After collaboratively concluding upon the themes as findings, we agreed that the midwifery students described MR as a *supplementary* learning method—not a full-blown replacement for classroom teaching, simulation, or clinical practice. Therefore, this is presented as an overarching theme, also described by one of the students as follows:

After receiving other information and attending lectures on it, I think it’s a nice supplement for learning. It also makes it a bit fun. But all in all, as a supplement to the knowledge I have gained, I think it’s nice. (Informant *8)*

## Discussion

The overarching finding from the analysis pointed towards MR as a *supplementary* learning method—not a replacement—which aligns with pedagogical assumptions by Biggs and Tang [46], who underscore that “all learning takes place against a backdrop of existing knowledge.” Similarly, Knowles, Holton [47] emphasises this engagement and to know your “why” in one’s own learning. Furthermore, the pedagogical potentials are supported in this study by focusing on communication, cooperation, and collaboration, illustrated in one of the sub-themes.

Students expressed MR as a valuable novelty related to individual learning, engagement, and collaboration. They described MR as engaging and entertaining, aligning with Sawyer’s [48] notion that deeper learning takes place more cognitively than traditional classroom services. It is worth noting that while entertainment value can be a desirable feature of certain lessons or learning paradigms, the positive engagement generated by or alongside novel or even “fun” lessons or teaching material can, if taken too far, counterproductively spill over into distraction or disengagement from the core didactic content.

While MR content can (and even should, by definition) appeal to sensory modalities beyond just hearing and vision, the foetal descent prototype evaluated in the present study lacked tactile feedback: The ability to perform digital cervical exam had not been pre-programmed into the lesson at this stage. Hence, one informant’s comments about a lack of haptics are appropriate. This student’s comments speak to a tension seen in VR research since the 1990s: The higher fidelity a representation, the more likely novice users are to expect to be able to touch it; to be able to interact with the virtual content as if tangible and real [49]. When a virtual or Mixed Reality representation does not conform to a user’s expectations (e.g. by being ethereal or non-tactile), this can result in a kind of immersion-breaking disengagement that may be harmful to a learner’s attention irrespective of the quality of the learning materials.

Related to the subtheme “An Engaging Bubble,” this speaks to the sense of presence mentioned in the paper’s Relevant Concepts and Similar Studies section, and to the related phenomenon of attentional engagement [33], as discussed earlier. That the giant foetal–uterine visualisation seemed believable to the eyes presumably contributed to it successfully capturing the students’ perceptual and cognitive attention. That the students were therefore not paying attention to their physical surroundings or interoceptive sensations (e.g. hunger, discomfort, boredom) means the lesson was *necessarily* more engaging than one that does not steal attention or engage the learner. Gesturing to the parent theme, “A Valuable Novelty,” we might note that the strong sense of presence and engagement routinely commanded by even unremarkable VR and MR content will likely not last forever. Just as early cinemagoers became accustomed or desensitised to film’s dazzling sights and sounds over the course of a couple of decades at the turn of the 20th century, VR and MR users will likely not be quite as captivated or mesmerised by immersive content in ten or twenty years as they are today.

Students mention that they learn better in collaboration with other students and lecturers. This, taken alongside the concept of presence as evidenced in the theme “An Engaging Bubble” align with Sawyer’s [48] view on pedagogy as he describes the educator as a scaffold-builder. Here, he argues that the pedagogue’s role is to support the students’ own learning and that the learning environment (inclusive of peers) constitutes the scaffolding that supports the construction of knowledge. These scaffolds should then be gradually modified based on the current needs as learning progresses and knowledge levels increase. This progression moves the teaching from concrete to more abstract, providing students with opportunities to utilise their early acquired surface knowledge and, through processes such as externalisation, articulation, and reflection, build deep learning [48]. Similarly, Hattie [50] mentions a corresponding progression when discussing “conceptual change.” Scaffolding generally denotes the provisional assistance given to help learners complete a task that they might not be able to finish on their own [51].

Teaching and learning methods intended to actively engage students are collectively referred to as student-active learning methods. This further aligns with how students in this study experienced engagement to facilitate learning. According to Geven and Attard [52], the meaning of the term is vague. Student-centred learning remains a debated concept for which there is no unambiguous definition. However, Freeman, Eddy [53] have proposed what they call a consensus definition of student-active learning. Their definition states that active learning involves students in the learning process through activities and discussions during class, rather than passively listening to an instructor. This approach emphasises higher-order thinking and frequently incorporates group work. The perspective traces back to Vygotsky and Piaget and the social constructivist episteme [51].

Research indicates that integrating relevant clinical examples with complex subjects not only enhances knowledge and understanding but also increases students’ awareness of the subject’s importance and relevance [41]. During the MR session, students collaborated in small groups, which was a deliberate pedagogical strategy aligned with deliberate practice described by Ericsson [54]. Recognising that foetal stations is a complex topic requiring reflection through discussion and explanation, this approach was designed to ensure high-quality interaction and in-depth learning within the groups. Students were paired with peers with whom they were already familiar and reported that small-group activities provided a safe environment for sharing knowledge. Working with peers proved to be more beneficial than working alone due to the subject’s complexity. The simultaneous interaction in the mixed reality classroom fostered a sense of togetherness, enhancing their understanding. Additionally, students noted that the teacher’s role as a discussion partner (rather than merely a transmitter of knowledge) facilitated effective knowledge exchange within the group. These experiences corresponded completely to Aasekjær, Gjesdal [55], whose research focused on learning anatomy through VR.

Repeated learning (also related to low-dose, high-frequency) in a peer-to-peer learning method has been used widely for decades within healthcare professions and simulation training [56]. As suggested by students in this study, small groups of four to six students create a safe learning environment. According to Jeffries [57], being confident in simulation scenarios is a prerequisite for effective learning. Here, we aim to draw parallels to Fossland’s [58] suggestion that educators should utilise technology beneficially for students, preparing them for future professional environments, and bridging clinical practice and theoretical education. It is well known that the healthcare sector employs simulation at many levels, incorporating varying degrees of technology for knowledge, innovation and skill maintenance [59]. Lastly, the results of our study align with Saljo [60], who argues that digital technologies transform how we learn and our interpretation of learning itself, which challenges traditional teaching roles and practices. This shift in perspective is consistent with observations in teaching and learning practices within higher education.

### Strengths and limitations

More than 12 students expressed interest in participating in interviews, but further interviews were not conducted due to scheduling constraints within the planned timeframe for the students. The teachers at USN were not committed to Laerdal, allowing them to conduct the interviews independently, which potentially facilitated students’ candid discussion of their negative experiences. The intervention involved a mixed reality session conducted in English, with a local teacher available in the room for translation if needed. Given that the students were Norwegian, the interviews were conducted in Norwegian to enable them to freely express themselves in their native language and minimize any discomfort in discussing negative experiences. Language barriers could have posed challenges for the students. A notable strength was the analysis conducted in collaboration with two midwifery educators to ensure a shared understanding of the data.

### Implications and recommendations

Further studies are welcomed to evaluate the use of MR technology in midwifery education, particularly for teaching students about foetal descent during labour. This study had a small sample size, so we recommend conducting further research with a larger group. Given the rapid advancements in the MR industry, the specific technology used may differ in future studies, although similar tools could be applied. Importantly, technology in midwifery education is not frequently utilised, so it is recommended that any tools adopted should be simple and easy to use.

## Conclusions

This qualitative study concludes that mixed reality (MR) is a valuable supplement to existing teaching methods in midwifery education. Students expressed optimism about MR’s potential to provide customisable lessons, captivate users, and foster discussion, collaboration, and productive debate. They appreciated the ability of MR lessons to be used repeatedly with optimised group sizes. While students found the technology intuitive, they emphasised the importance of aligning learning content with students’ existing knowledge and abilities. MR was perceived as a desirable and effective supplementary learning tool. While it cannot replace dialogue with a qualified instructor, it is well-suited to peer-to-peer learning. MR can offer richer, higher-fidelity representations of anatomy and physiology than can physical manikins alone. We recommend integrating multiple learning methods into a given MR-enhanced curriculum to enhance educational outcomes. As future studies are conducted to objectively evaluate the impact of MR on learning, it is important to recognise that MR should not replace humanistic pedagogy, but rather serve to augment it.

## Data Availability

The dataset used and analysed during this study is available from the corresponding author upon reasonable request.

## Abbreviations

LDHF: Low-dose high-frequency
MR: Mixed Reality
UX: User experience
VR: Virtual Reality
